# A quasi-experimental study to mobilize rural low-income communities to assess and improve the ecological environment to prevent childhood obesity

**DOI:** 10.1186/s12889-016-3047-4

**Published:** 2016-05-04

**Authors:** Paula Peters, Abby Gold, Angela Abbott, Dawn Contreras, Ann Keim, Renee Oscarson, Sandra Procter, Valentina Remig, Carol Smathers, Amy R. Mobley

**Affiliations:** Kansas State Research & Extension, Kansas State University, 340 Justin Hall, Manhattan, KS 66506 USA; Department of Public Health, North Dakota State University, Dept 2662, PO Box 6050, Fargo, ND 58108-6050 USA; Purdue University Extension, Matthews Hall, 812 W. State Street, West Lafayette, IN 47907 USA; Michigan State University Extension, 160 Agriculture Hall, East Lansing, MI 48824 USA; University of Wisconsin Extension, 432 N. Lake Street Room 631, Madison, WI 53706 USA; South Dakota State University, Box 2275A, Brookings, SD 57007 USA; The Ohio State University Extension, 381 Campbell Hall, Room163A, 1787 Neil Avenue, Columbus, OH 43210-1220 USA; Department of Nutritional Sciences, University of Connecticut, 3624 Horsebarn Road Extension, Unit 4017, Storrs, CT 06269 USA

**Keywords:** Low income population, Rural community, Pediatric obesity, Community networks, Community coaching

## Abstract

**Background:**

The Ecological Model of Childhood Overweight focuses on characteristics that could affect a child’s weight status in relation to the multiple environments surrounding that child. A community coaching approach allows community groups to identify their own strengths, priorities and identity. Little to no research currently exists related to community-based efforts inclusive of community coaching in creating environmental change to prevent childhood obesity particularly in rural communities.

**Methods:**

A quasi-experimental study will be conducted with low-income, rural communities (*n* = 14) in the North Central region of the United States to mobilize capacity in communities to create and sustain an environment of healthy eating and physical activity to prevent childhood obesity. Two rural communities within seven Midwestern states (IN, KS, MI, OH, ND, SD, WI) will be randomly assigned to serve as an intervention or comparison community. Coalitions will complete assessments of their communities, choose from evidence-based approaches, and implement nutrition and physical activity interventions each year to prevent childhood obesity with emphasis on policy, system or environmental changes over four years. Only intervention coalitions will receive community coaching from a trained coach. Outcomes will be assessed at baseline, annually and project end using previously validated instruments and include coalition self-assessments, parental perceptions regarding the built environment, community, neighborhood, and early childhood environments, self-reflections from coaches and project staff, ripple effect mapping with coalitions and, final interviews of key stakeholders and coaches. A mixed-methods analysis approach will be used to evaluate if Community Coaching enhances community capacity to create and sustain an environment to support healthy eating and physical activity for young children. ANOVA or corresponding non-parametric tests will be used to analyze quantitative data relating to environmental change with significance set at *P* < .05. Dominant emergent themes from the qualitative data will be weaved together with quantitative data to develop a theoretical model representing how communities were impacted by the project.

**Discussion:**

This project will yield data and best practices that could become a model for community development based approaches to preventing childhood obesity in rural communities.

## Background

Approximately one in four preschool age children are considered overweight or obese with higher rates among low-income families [1]. Four years of age appears to be a critical time in overweight and obesity prevention especially among children with a lower socioeconomic status [2]. An obese four-year-old child has a 20 % likelihood of being an overweight adult [3]. Prevention efforts for childhood obesity should be multi-faceted, involving children of all ages throughout all sectors of the environment. Accordingly, the U.S. White House Task Force on Childhood Obesity Report called for multi-sector collaborations to prevent childhood obesity [4].

The Center for Study of Rural America reports that people in rural areas suffer the highest obesity rates in the United States [5]. The geography and infrastructure of rural areas contribute to problems of poor nutrition and physical inactivity among rural populations. Specifically, children living in rural areas in the U.S. are about 25 % more likely to be overweight or obese than their urban counterparts [6]. One theory is that children living in rural areas have fewer chances than urban children for physical activity in their daily routine. While most states have obesity prevention plans, few plans seem to focus on the rural population and their special needs [7].

The Ecological Model of Childhood Overweight focuses on those characteristics that could affect a child’s weight status in relation to the multiple environments surrounding that child [8]. This model examines the combined effects of the community at large, parenting and family factors, and individual factors that impact a child’s weight. The Ecological Model considers the larger community in which the child lives. Consensus is building among researchers that the obesity epidemic is driven by the environment, rather than solely by individual factors [9–13]. Therefore, a closer examination of the community in regard to childhood obesity prevention is warranted.

Experience in community development reinforces the conclusion that work in this aspect must be locally conceived, locally led, and consistent with the cultural identity of the community [14]. A community coaching approach allows community groups to identify their own strengths, priorities and identity. Thus, community coaches may assist communities with an assessment of their strengths and weaknesses, and then engage the community in investigating new ways of solving their problems. Community coaches help the group strategize and gain commitment to move forward and may help groups use the knowledge and understanding they have gained to take their work to a higher level [15]. While information and program ideas are widely available on the internet, information alone does not create change. A community coach could help motivate communities to translate research and information into practice.

To address the issue of childhood obesity in rural communities, a collaborative, multi-state (Indiana, Kansas, Michigan, North Dakota, Ohio, South Dakota, and Wisconsin) effort was formed. Team members include nutrition scientists, community development specialists, and family and youth development specialists from the North Central region of the United States who developed an innovative, integrated research and Extension project. The overall goal of this project is to mobilize capacity in low-income, rural communities to create and sustain an environment and culture of healthy eating and physical activity to prevent childhood obesity in young children.

The specific project objectives are:

1. Existing health related coalitions in 14 rural U.S. communities (two per state, one as an intervention community and one as a comparison community) will be supported to improve the environment for nutrition and physical activity of 4‐year‐old children in their community.
2. The project team will identify or develop community assessment tools and assemble them into a *Child Ecological Model Assessment Tool Kit.*
3. The project team will create a menu of evidence‐based or evidence‐informed educational programs and protocols addressing healthy eating and physical activity for 4‐year‐old children from which communities will select.
4. Community coalitions will create a plan to reduce obesity in their community that is based on the assessment of their community’s environment for 4‐year‐old children.
5. Community coalitions will identify and implement at least one local or institutional policy, system or environmental change that will improve nutrition and one that will increase physical activity for 4‐year‐old children.
6. The project team will assess the perceived value that the community coalition members place on the effectiveness of the menu of evidence‐based or evidence informed educational programs and protocols in improving the environment that sustains healthy eating and promotes physical activity for 4‐year‐old children.
7. The project team will test the main hypothesis that Community Coaching will enhance the capacity of the community in preventing childhood obesity by addressing the needs identified in the assessment and by providing the community coalitions in the intervention community (one per state) with Community Coaches training.

To summarize, existing community health coalitions will assess their environment using an ecological model of childhood overweight and then select evidence-based or evidence-informed approaches to implement over four years to address their community needs. This research will examine the effectiveness of a community coaching model on the ability of a community coalition to address the needs identified to improving the healthy eating and physical activity environment for 4‐year‐old children.

## Methods

### Study design

The focus of this project is to improve understanding of those factors necessary to mobilize communities to make policy, system or environmental changes aimed at the prevention of childhood obesity within seven North Central U.S. States (IN, KS, MI, ND, OH, SD, WI). Using a quasi-experimental design, the research will examine how building community capacity with a community coaching model in rural, low-income communities contributes to the communities’ increased ability to prevent childhood obesity. The Ecological Model of Childhood Overweight will serve as the foundation for reviewing characteristics that could affect a child’s weight status in relation to the multiple environments surrounding that child [8].

Low-income, rural communities will be recruited to apply for the project from each of the seven participating states. Rural areas will be defined based on the Office of Management and Budget’s definition, which delineates an area as rural if there is no presence of a metropolitan statistical area or a core urban area with a population of 50,000 or more [16]. Low-income will be defined based on the community’s average income rate being below the state’s average poverty rate. Other criteria will include that the community has an existing community health coalition and community focus to tackle childhood obesity prevention. Similar-size intervention and comparison communities will be selected from each state. Once selected through an application screening process to ensure that selection criteria are met, communities will be randomly assigned to the intervention or comparison group. Each state will have one intervention and one comparison community. The project sample size (*n* = 14) will be sufficient to detect large effect sizes (d ≥ 0.8) with 95 % power for a two-tailed test at alpha = .05 between intervention and comparison communities from pre to post using the community as the main unit of measure and community level variables as the main outcomes.

### Intervention

Each community will utilize the efforts of a community health coalition to develop a strategic plan and to implement that plan after completing a series of assessments. Communities will be provided with a “menu” of evidence-based or evidence-informed strategies and a set amount of funding each year ($5000) to implement at least one nutrition and one physical activity intervention annually. The menu of evidence‐based or evidence‐informed childhood obesity prevention approaches will be developed by the research team and continually updated and, made available electronically to both intervention and comparison communities. Materials selected will be culturally and ethnically diverse, representing the varied ethnic groups that make up the North Central U.S. region, including Caucasian, Hispanic, Native American, Hmong, and other groups. The menu will include evidence-based nutrition and physical activity curricula, evidence‐based or evidence‐informed strategies as well as recommendations from the U.S. White House Task Force on Childhood Obesity Report to the President [4]. Nutrition interventions will seek to improve the environment within the communities to support an increased intake of fruits and vegetables, increase the variety of vegetables in the diet, or decrease the intakes of foods high in solid fats and added sugars while physical activity interventions will seek to increase the number of children that meet the guidelines for television viewing and computer use, or increased physical activity.

The intervention communities will also be provided with a part-time community coach for the full length of the project. Responsibilities of the coach will be to guide the coalition in identifying, clarifying, and illuminating local childhood obesity issues, goals, prevention strategies, partners and resources. The comparison communities will not be provided with a coach and on their own will choose and implement nutrition and physical activity approaches throughout the duration of the project. A study timeline is provided in Table 1.

**Table 1:** Project timeline and milestones

Year	Activities	Milestones
1	• Hire program coordinator	• Staff hired
	• Identify, adapt, and/or develop the Child Ecological Model Assessment Tool Kit	• Tools developed
	• Develop “menu” of selected evidence based or evidence‐informed approaches for improved nutrition and physical activity for 4‐year‐olds	• Communities chosen
	• Choose intervention and comparison communities	
2	• Conduct community assessments	• Child Ecological Model Assessments completed
	• Hire community coaches for intervention communities	• Coaches hired and trained
	• Train community coaches and other key leaders of intervention communities on community coaching	• Interventions implemented
	• Each of the intervention and comparison communities begin implementation of at least one nutrition and one physical activity intervention	
3	• Interventions continue	• Interventions implemented
	• Training continues	• Process evaluations monitor progress of interventions
	• Process evaluations on‐going	
4	• Interventions continue	• Interventions implemented
	• Training continues	• Process evaluations monitor progress of interventions
	• Process evaluations on‐going	
5	• Interventions continue for final year	• Interventions implemented for final year
	• Intervention communities develop sustainability plan	• Data analysis completed and disseminated
	• Complete post‐assessments	• Tool kit and other findings available on‐line
	• Analyze data	
	• Organize findings into a “Best Practices Tool Kit” for dissemination via technology such as eXtension	
	• Research manuscripts and conference proposals begin	

Each state has secured the necessary Institutional Review Board (IRB) approval or exemptions as required by their institution. For the main study intervention where the community was the unit of randomization and intervention, approvals were secured by The Ohio State University Behavioral and Social Sciences IRB and the Michigan State University IRB. Study exemptions were secured by the Kansas State University Research Compliance Office, North Dakota State University IRB, Purdue University Human Research Protection Program IRB, South Dakota State University Office of Research Human Subjects Committee and, University of Wisconsin Extension Human Subjects Protection Committee. When outcomes involved assessment or interviews with human subject participants related to the community interventions, informed written consent was obtained (Ohio State University) or information sheets were provided to participants (North Dakota State University) when required by specific institutional IRB.

### Measures

A summary of measures is included in Table 2.

**Table 2:** Summary and timeline of outcome measures for intervention and comparison communities

Measure/ variable	Target audience	Tool	Year 1	Year 2 (PRE)	Year 3	Year 4	Year 5 (POST)
Coalition self-assessments	Community coalition members			x	x	x	x
Parental perceptions and practices	Low-income parents of preschool age children living in project communities	Active Where [17]		x			x
Community-at-large environment	Project communities	CHLI [18]		x			x
Neighborhood environment	Project community neighborhoods	CHLI [18]		x			x
Early Childhood Center policies and practices	Project community early childhood centers	CHLI [18]		x			x
Project Reflections	Project staff, coaches			x	x	x	x
Interviews	Coalition members, coaches						x
Ripple effect mapping	Community coalition members						x

*CHLI* Community Healthy Living Index

### Coalition Self-Assessment Survey (CSAS)

The CSAS will be used to assess coalition development, functioning and effectiveness. It contains six different domains of measurement: Environmental Characteristics (of the coalition), Structural Characteristics (of the coalition), Functional Characteristics (of the coalition), Coalition Programs and Interventions, Intermediate Measures of Coalition Effectiveness, and Outcome Measures of Coalition Effectiveness. This tool will be administered annually to each of the states’ community health coalition members to serve as an evaluative measure for assessing function, process, and outcomes of the coalitions; and to provide quantitative information on the progress of the coalitions throughout the span of the project.

### Active where?

The Active Where? Parent Survey will be used as a quantitative assessment of parent perceptions of the built environment. This survey will be adapted, with permission, from the Active Where? Parent-child Survey [17] to contain a demographic section and 11 sections to assess home and neighborhood environment characteristics including: recreation and sports facilities where your child plays; barriers to activity in the neighborhood; access to services; neighborhood streets; places for walking/biking; neighborhood surroundings; neighborhood safety; local environment; physical activity; rules for eating; and food. Questionnaire sections with Cronbach α < .70 will be examined for low scoring items and, items will be deleted to improve reliability. Content and face validity will be established by the project research team.

A total of 30 participants will be recruited from each of the rural, low-income target intervention (*n* = 7) and comparison communities (*n* = 7) to complete the Active Where? questionnaire. Active Where? will be administered at baseline and in the final year of the project. Eligibility criteria for each participant will include: being at least 18 years of age and a parent or legal guardian of a child between the ages of 3 and 5 years, ability to speak and/or read English, being a resident of the target community, and being enrolled or having a child enrolled in programs such as Head Start, SNAP (Supplemental Nutrition Assistance Program) or WIC (Special Supplemental Nutrition Program for Women, Infants and Children). The interviewer will read the consent form or information sheet to the participants and allow them to ask questions before beginning the survey interview. Using a standard protocol, questionnaires will be administered face-to-face at the site of recruitment and each parent participant will complete the survey once. It is estimated that each interview will take approximately 45–60 min. Participants will be given a small monetary incentive after completing the interview.

### Community Healthy Living Index (CHLI)

The YMCA’s Community Healthy Living Index (CHLI) assessments will be used in each of the project communities [18]. The CHLI contains assessments for six key community settings: afterschool child care sites, early childhood programs, neighborhoods, schools, work sites, and the community at large. Each assessment contains questions about policies and practices that support healthy lifestyles. Three of the six assessments will be selected (early childhood programs, neighborhood and community at large) for the project as they specifically support the project objectives. Assessments will be conducted by at least one individual in the community health coalition at baseline, and follow-up assessments will be conducted in the final year of the project. During the first year of data collection, communities will utilize the results of the CHLI tools to identify areas that need improvement within the various settings and plan for action to improve the ecological environment of their community to ultimately prevent childhood obesity.

### Qualitative data

Throughout the project, each state’s co-investigator, graduate student and community coach will complete monthly or quarterly reflections to capture progress, impressions and potential changes over the course of the research. At the end of the project, community coalition member and coach one-on-one interviews will provide additional focused qualitative information. Reporting on qualitative data collection will adhere to the consolidated criteria for reporting qualitative studies (COREQ) guidelines [19].

To assess the value of the menu of evidence-based or evidence informed educational programs, interviews will be conducted with coalition members of both the intervention and control communities. The interviews will be conducted by trained staff, recorded and transcribed for analysis. Participants will be asked their opinions about the usefulness and quality of the items in the menu of intervention strategies that were provided to them.

### Ripple effect mapping

Ripple effect mapping (REM) is a mind mapping technique used to examine how establishing natural and built assets can serve as a catalyst to leverage other community assets. REM is a promising way of measuring impact that engages stakeholders and program participants. Hanson and Kollock state, “REM is best conducted for in-depth program interventions or collaborations that are expected to produce broad or deep changes in a group, organization, or community” [20]. The emergence of new organizations, institutions, programs, policies, or strategies related to the intervention will be studied. The emergent support of organizations and institutions to sustain interventions as a broader community development “spiraling up” will be noted [21]. REM will be used in both intervention and comparison communities using a standard protocol and specialized mind mapping software [22]. A research team from each state will conduct ripple effect mapping with the two community coalitions in their state after the coalitions complete their final interventions.

### Statistical analysis

Objectives 1–5 are project development or process objectives which will be evaluated using monthly or annual reports from each state during the five year project. Objective 6 (perceived value of the menu of evidence-based obesity prevention resources) will be evaluated by qualitative data analysis techniques specifically, interviews with community coalition members. The main project objective, objective 7 (enhancement of community capacity to prevent childhood obesity by Community Coaching) will be evaluated using a mixed-methods data analysis approach with data from several of the project instruments (CHLI, Active Where?, and CSAS). Descriptive statistics including frequencies and repeated measures ANOVA or corresponding non-parametric tests will be conducted to detect changes between intervention and comparison communities before and after the four year intervention for quantitative data related to policy, systems and environmental change variables using SAS 9.4 software (SAS Institute Inc.). Significance will be set at *P* < .05.

Qualitative data (reflections and interviews) will be analyzed using the classic analysis approach [23] including constant comparison analysis to generate themes and search for text that inform those themes. Ripple effect mapping will be analyzed through content analysis by categorizing identified community activities and changes into the different levels of the social-ecological model. Dominant emergent themes from the qualitative data will be weaved together with quantitative data to develop a theoretical model representing how communities were impacted by the project.

### Study status

The intervention was implemented within rural communities from 2012–2016. The study is currently ongoing with a no-cost funding extension to finalize data collection and begin data analysis of main study outcomes.

## Discussion

As a result of the study, a comprehensive childhood obesity prevention *Best Practices Tool Kit* will be developed to assist low‐income rural communities to 1) assess the environments of their communities as they relate to childhood obesity prevention and 2) learn to use community coaching techniques to help communities assess their strengths, strategize, and implement interventions to prevent childhood obesity.

Strengths of the study include the involvement of seven U.S. states in a new model (community capacity development using community coaching) of Extension intervention to prevent childhood obesity; a rural community setting; use of an ecological model of childhood overweight to focus on the environment of low‐income, 4‐year‐old children; and involvement of a community coalition to identify their own needs and implement corresponding intervention activities. Having communities choose their own interventions may increase the likelihood of sustainability once the project is complete. Limitations of the study include the inability to control for external factors which may contribute to or deflect from the development of community networks and collaborative local action around the issue of childhood obesity prevention; inability to identify identical communities within each state for comparison purposes and the relatively short time frame in which to measure and expect environmental and policy changes. Lessons learned from this integrated, community-based study will demonstrate an innovative way that Extension can work within communities to drive change especially given the emphasis on Health Extension as the next frontier.

### Ethics approval and consent to participate

Each state has secured the necessary Institutional Review Board (IRB) approval or exemptions as required by their institution. For the main study intervention where the community was the unit of randomization and intervention, approvals were secured by The Ohio State University Behavioral and Social Sciences IRB and the Michigan State University IRB. Study exemptions were secured by the Kansas State University Research Compliance Office, North Dakota State University IRB, Purdue University Human Research Protection Program IRB, South Dakota State University Office of Research Human Subjects Committee and, University of Wisconsin Extension Human Subjects Protection Committee. When outcomes involved assessment or interviews with human subject participants related to the community interventions, informed written consent was obtained (Ohio State University) or information sheets were provided to participants (North Dakota State University) when required by specific institutional IRB.

### Availability of data and materials

Final raw data files will not be available in a public repository because it is not a requirement of the funding agency. Resulting reports and materials including a best practices toolkit will be available upon request by contacting the lead author (PP).

## Abbreviations

CHLI: Community Healthy Living Index
CSAS: coalition self-assessment survey
IRB: Institutional Review Board
REM: ripple effect mapping
SNAP: Supplemental Nutrition Assistance Program
WIC: Special Supplemental Nutrition Program for Women, Infants and Children
